# The Impact of Light-Curing Time on Shear Bond Strength and Adhesive Remnant Index (ARI)

**DOI:** 10.3390/jcm15051890

**Published:** 2026-03-02

**Authors:** Mahmoud Elsaafin, Clara Diana Haddad, Tamara Rahela Ioana, Marioara Moldovan, Mihai Vlad Golu, Valeriu Mihai But, Sorana Nicoleta Roșu

**Affiliations:** 1 Department of Orthodontics, Faculty of Dentistry, George Emil Palade University of Medicine, Pharmacy, Science and Technology, 540139 Targu Mures, Romania; nabil.elsaafinmahmoud@umfst.ro; 2 Department of Orthodontics, Faculty of Dentistry, Grigore T. Popa University of Medicine and Pharmacy, 700115 Iasi, Romania; haddad.claradiana95@gmail.com; 3 Department of Orthodontics, Faculty of Dental Medicine, University of Medicine and Pharmacy of Craiova, 200349 Craiova, Romania; tamigmg@yahoo.com; 4 Institute of Research in Chemistry Raluca Ripan, Babes-Bolyai University Cluj-Napoca, 30 Fantanele Street, 500327 Cluj-Napoca, Romania; mmarioara2004@yahoo.com; 5 Department of Oral and Maxillofacial Surgery, George Emil Palade University of Medicine, Pharmacy, Science and Technology, 540139 Targu Mures, Romania; vlad.golu@umfst.ro; 6 Department of Medicine-Psycho-Neuroscience and Recovery, Medical Oncology, Faculty of Medicine and Pharmacy, University of Oradea, 410073 Oradea, Romania; dr.butvaleriu@gmail.com; 7 Department of Oral and Maxillofacial Surgery, Faculty of Dentistry, Grigore T. Popa University of Medicine and Pharmacy, 700115 Iasi, Romania

**Keywords:** orthodontic brackets, shear bond strength, light curing, LED, adhesive remnant index, orthodontic bonding, enamel integrity

## Abstract

**Background/Objectives**: The performance of the adhesive system used for bonding fundamentally influences the success of fixed orthodontic treatment. To withstand masticatory forces and prevent bracket debonding, it is critical to achieve optimal shear bond strength (SBS), improving treatment time. The aim of this study was to compare the shear bond strength and adhesive remnant index (ARI) of orthodontic brackets bonded using LED light-curing units with intensities of 3200 mW/cm^2^ at two different exposure times, evaluated after 24 h and 14 days. **Methods**: Eighty extracted permanent premolars were randomly divided into four experimental groups. Brackets were bonded using Transbond XT adhesive and cured with a VALO™ Ortho Cordless unit (3200 mW/cm^2^) for 3 or 6 s. SBS was measured using a universal testing machine, and the ARI was assessed under a stereomicroscope at 40× magnification. **Results**: Increased light intensity and longer curing time significantly improved SBS values. The highest bond strengths were observed in groups cured with 3200 mW/cm^2^ for 6 s, after 14 days. ARI scores showed that longer curing reduced adhesive remnants on the enamel. Statistical analysis confirmed significant differences among groups. **Conclusions**: Therefore, clinicians may achieve better bonding performance and easier enamel clean-up by using high-intensity lights with adequate curing duration.

## 1. Introduction

In the field of orthodontics, successful treatment outcomes depend, among other primordial factors, on the proper adhesion of the bracket to the enamel. This implies that brackets should withstand masticatory forces, which requires the adhesive system to be resilient and to resist failure even when exposed to great forces directed at the bracket. This resistance is measured by in vitro shear bond strength (SBS). Otherwise, when a bracket is loosely bonded, it might break or dislodge, which leads to additional treatment time for both the clinician and the patient. Apart from the matter of time, this scenario also involves supplementary financial implications and negative consequences related to the enamel surface, since the clinician must remove the resin prior to bonding a new bracket [1,2,3]. Moreover, fixed orthodontic appliances are associated with an increased risk of incipient carious lesions, commonly referred to as white spot lesions (WSLs). They are one of the most prevalent complications in patients undergoing orthodontic treatment and are primarily caused by enamel demineralization resulting from plaque accumulation over time in the area surrounding the brackets, as well as adhesive materials [1,2,3].

However, once the orthodontic treatment is considered complete, the next step for the orthodontist is the removal of the adhesive left after debonding. During this stage, a very important aspect is to try to cause as little damage as possible to the enamel while making sure to remove all of the adhesive from the teeth. However, the clinician might find it necessary to compromise and leave a small amount of adhesive behind. One of the reasons for this is the color of the adhesive, which is very similar to the color of the tooth. The amount of adhesive remaining on the enamel is evaluated using the adhesive remnant index (ARI), quantifying the proportion of resin left on the tooth surface following debonding. Importantly, effective light curing of the adhesive is not only about securing the bracket in place but is also critical in ensuring the complete polymerization of the adhesive resin. Therefore, incomplete polymerization may result in greater volumes of residual adhesive that are more difficult to remove. This represents the beginning of two primary critical challenges. First, excessive residual adhesive creates retentive areas on the enamel surface, facilitating biofilm accumulation, which can lead to decalcification and the development of WSLs. The second problem associated with residual adhesive involves the pigmentation of adhesive remnants caused by aging [4].

In this context, previous studies on resin-based dental materials have shown that prolonged exposure to acidic and staining agents can induce surface degradation, microhardness reduction, and chemical alterations within the polymer matrix. Such changes may increase surface roughness, promote discoloration, and favor plaque retention, ultimately compromising the long-term clinical performance of resin materials [5].

Light-emitting diode (LED) systems have proven to be an efficient approach to curing orthodontic adhesives. Due to their wide field of applications, light-curing units are now indispensable for orthodontists and general dentists. As mentioned before, in orthodontics, it is crucial to achieve the optimum bond strength between the bracket and the tooth surface, which is why LED technology is preferred for the polymerization of light-initiated dental materials. The mechanical properties of composite resin depend on an optimal degree of cure, which is directly related to the intensity of light and radiation exposure time [4,6,7,8].

In recent years, manufacturers of high-intensity LED curing units have claimed that their devices are able to effectively bond within curing times as short as 3 s. However, despite these claims, the ability of such ultra-short curing protocols in achieving bond strength and degree of polymerization compared to conventional, longer curing times remains uncertain. Insufficient curing may not only compromise bracket retention but may also lead to incomplete adhesive polymerization, increasing the risk of adhesive remnants and the associated complications of plaque accumulation and WSL formation [4,6,7,8].

WSLs, commonly referred to as incipient carious lesions, are common complications found in fixed-orthodontics patients. These are caused primarily by etching the surface of the enamel, as well as by poor oral hygiene and the accumulation of microbial biofilms during orthodontic treatment [9]. Given the widespread adoption of high-intensity LED curing units and the trend toward reducing curing times in clinical orthodontic practice, there is a need to evaluate whether ultra-short curing protocols can achieve adequate bond strength without compromising adhesive polymerization and, consequently, enamel safety. Specifically, it remains unclear whether a 3 s curing time produces shear bond strength and adhesive remnant outcomes comparable to those of longer, conventional curing protocols. Understanding this relationship is of significant clinical relevance as it has direct implications for bracket retention, the efficiency of the debonding procedure, and the risk of WSL development associated with residual adhesive on the enamel surface.

Therefore, the aim of this in vitro study was to evaluate and compare the shear bond strength and adhesive remnant index of orthodontic brackets cured with a high-intensity LED unit at different curing times, in order to determine whether ultra-short curing protocols can produce results comparable to conventional protocols. The null hypothesis was that there is no statistically significant difference in shear bond strength or adhesive remnant index between the ultra-short (3 s) curing protocol and conventional (longer) curing protocols.

## 2. Materials and Methods

This study was conducted on 80 extracted permanent premolars, which were free of restorations, caries, fractures, or wear. Extracted teeth were obtained after routine therapeutic procedures with patients’ informed consent for research use. Samples were anonymized prior to laboratory handling. To prevent the enamel surface from dehydration, all extracted premolars were stored in normal saline solution, which was replaced weekly with fresh solution to avoid bacterial growth. All selected teeth were polished with fine pumice to clean their enamel surface.

Phosphoric acid (Blue Etch) with a 36% phosphoric acid concentration was used to etch the enamel surface, followed by 15 s washing with a water spray. After that, the enamel surface was dried for 5 s with oil-free compressed air.

The enamel of the teeth was primed with adhesive primer (Transbond XT Primer, Ultradent Products, Inc., South Jordan, UT, USA). With the aid of a positioning gauge, brackets (Unitek TM Miniature Twin Metal Brackets, 3M Unitek Orthodontic Products, Monrovia, CA, USA) were then bonded in the proper position (4 mm from the occlusal surface of the teeth), using a small amount of 3M’s adhesive material (Transbond XT Primer, Ultradent Products, Inc., South Jordan, UT, USA and Transbond adhesive paste material, Ultradent Products, Inc., South Jordan, UT, USA).

Prior to light curing, the excess adhesive material was removed using a sharp probe while keeping the brackets in their original positions. After bonding the brackets, each tooth was mounted in cold-cure acrylic as a base, which was made by mixing and pouring the acrylic into a polypropylene pipe ring. The teeth were embedded vertically inside the self-cured acrylic resin blocks before they set. The labial surfaces of teeth were positioned at least 2 mm above the top surface of the acrylic resin after bonding the brackets.

Depending on the experimental group, the adhesive material was light-cured using VALO™ Ortho Cordless light cure Xtra Power (Ultradent Products, Inc., South Jordan, UT, USA): 3200 mW/cm^2^ for either 3 s (vestibular side) or 6 s (3 s on the mesial side and 3 s on the distal side). The eighty teeth were randomly divided into 4 experimental groups.

For the first and third groups, the light curing was performed for 3 s from the occlusal aspect, positioning the light-guide tip as close as possible to the bracket.

For the second and fourth groups, the light curing was performed for 6 s from the mesial and distal directions. The light-guide tip was positioned at a 45-degree angle to the tooth surface.

Group 1: Three seconds of light curing (VALO™ Ortho Cordless, 3200 mW/cm^2^), followed by testing after twenty-four hours.

Group 2: Six seconds of light curing (VALO™ Ortho Cordless, 3200 mW/cm^2^), followed by a 24 h testing period.

Group 3: Three seconds of light curing (VALO™ Ortho Cordless, 3200 mW/cm^2^), followed by 14-day testing.

Group 4: Six seconds of light curing (VALO™ Ortho Cordless, 3200 mW/cm^2^), followed by 14-day testing.

The difference in curing direction between groups was required in order to respect the manufacturer’s recommended protocol for each curing time. The 3 s curing protocol (Groups 1 and 3) was designed to be applied as a single exposure from the occlusal aspect, with the light-guide tip positioned as close as possible to the bracket, in accordance with the VALO™ Ortho manufacturer’s instructions for the Xtra Power mode. For the 6 s protocol (Groups 2 and 4), according to the manufacturer’s recommended technique for ensuring adequate light propagation around the bracket base, total curing time was divided into two exposures, each lasting 3 s and delivered from the mesial and distal aspects at a 45-degree angle. It is acknowledged that this variation in curing direction introduces a potential unwanted variable, as light angulation may affect the distribution and degree of polymerization across the adhesive layer. This methodological aspect is further addressed in Section 5.

Prior to testing the SBS, the premolars with brackets were preserved at 37 °C in artificial saliva. The shear bond strength (SBS) test was assessed using Lloyd LR5k Plus testing machine (Ametek Lloyd Instruments, Meerbusch, Germany). Occluso-cervical force was delivered to the adhesive-bracket interface at 1 mm per minute. The shear bond strength measurement was used to convert the observed forces at the bracket’s surface from Newtons (N) to Megapascals (MPa). This relationship is expressed mathematically as follows: SBS = F/A (MPa or N/mm^2^), where F is the detachment force in newtons and A is the bracket base surface area. After debonding, the adhesive remnant indices (ARI) scores were assessed under a stereomicroscope at 40× magnification.

The adhesive remnant indices (ARI) were categorized using a scale ranging from 0 to 3, according to the Artun and Bergland scale.

0 = no adhesive material remains on the surface of the premolars.

1 = fewer than 50% of the adhesive material remains on the premolar surface.

2 = more than 50% of the adhesive material remains on the surface of the premolars, but less than 100%.

3 = 100% of the adhesive material remains on the surface of the premolars.

Statistical analysis employed one-way analysis of variance (ANOVA) and the Tukey post hoc test for SBS comparisons. However, the Kruskal–Wallis test was used to compare the groups of ARI values. Statistical significance was set at *p* < 0.05. Eleven statistical analyses were performed using IBM SPSS Statistics version 23.0 (IBM Corp., Armonk, NY, USA), with the significance level set at *p* < 0.05.

## 3. Results

### 3.1. Shear Bond Strength

This study compared four groups based on curing time (3 vs. 6 s) and testing time (24 h vs. 14 days). Significant differences (*p* < 0.05) were found between most of the groups. Groups 1, 2, 3, and 4 (using the 3200 mW/cm^2^ light source) displayed statistically significant differences between each group, except between Groups 3 and 4 (*p* = 0.405). This indicates that, at 14 days, increasing the curing time from 3 to 6 s did not result in a significant increase in shear bond strength (SBS) for the 3200 mW/cm^2^ unit. Overall, the groups tested after 14 days tended to show higher SBS, suggesting that bond strength continues to develop over time due to ongoing polymerization.

**Figure 1:**
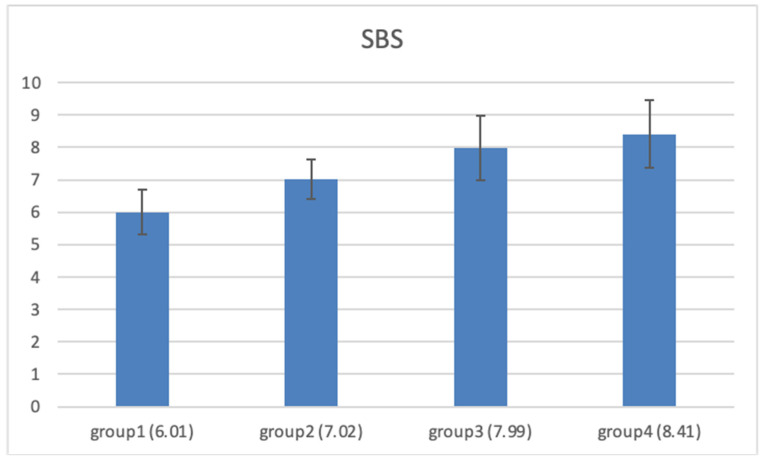
Mean shear bond strength (SBS) values (MPa) for each experimental group (*n* = 20 per group). Error bars represent the standard deviation.

The following section presents the mean shear bond strength (SBS) values (MPa) for each experimental group (*n* = 20), illustrated in Figure 1 with error bars representing the standard deviation, along with the pairwise comparisons between groups using the Tukey post hoc test (*p*-values), summarized in Table 1.

### 3.2. Adhesive Remnant Index

As shown in Table 2, according to the Kruskal–Wallis test (*p* = 0.001, Chi-Square = 9.543), significant differences in ARI scores existed among groups. The frequency distribution of ARI scores revealed that Group 1 (3 s, 3200 mW/cm^2^, 24 h) had the highest proportion of specimens with scores of 2 and 3 (30% and 25%, respectively), indicating that more than half of the specimens (55%) retained over 50% of the adhesive on the enamel surface, while only 15% showed no adhesive remnants (score 0). Group 2 (6 s, 3200 mW/cm^2^, 24 h) displayed a similar pattern, with scores 1 and 2 each accounting for 35% of specimens, although the proportion of score 3 decreased to 10%, suggesting a modest improvement in adhesive removal with a longer curing time at the 24 h time point. Group 3 (3 s, 3200 mW/cm^2^, 14 d) showed a notable shift toward lower ARI scores, with 30% of specimens presenting with score 0 and 40% presenting with score 1, while scores 2 and 3 each accounted for only 15%. This trend was most pronounced in Group 4 (6 s, 3200 mW/cm^2^, 14 d), which had the highest proportion of score 0 (45%) and score 1 (40%), with only 15% of specimens at score 2 and no specimens receiving score 3 (0%). This progressive shift from higher to lower ARI scores across groups indicates that both longer curing times and extended post-bonding aging were associated with reduced adhesive remnants on the enamel surface.

The Kruskal–Wallis test showed that the differences were statistically significant (*p* = 0.001), confirming that the curing method influences the amount of adhesive left on the teeth.

These results support the concept that using a more powerful light (3200 mW/cm^2^) and a longer curing time (6 s) can lead to higher bond strength and more efficient adhesive removal. From a clinically oriented perspective, curing time and light intensity significantly affect both bond strength and residual adhesive. Hence, a lower ARI score means easier enamel clean-up after bracket debonding.

## 4. Discussion

One of the most dynamic fields in orthodontic materials research is the assessment of the adhesive bond strength between a bracket and etched tooth enamel or other surfaces. The shear bond strength of clinically effective composites when bonded to human enamel and dentin has been reported to range from 5.9 MPa to 7.8 MPa [10]. Moreover, intraorally, bonded brackets are subjected to various forces, and bond strength can be influenced by several factors, including the type of light-curing equipment, the kind of enamel conditioner, the acid concentration, the etching duration, the adhesive’s composition, the bracket base design, and the material of the bracket [11,12,13,14,15,16,17].

However, the null hypothesis of this study stated that there is no statistically significant difference in shear bond strength or adhesive remnant index between the ultra-short (3 s) curing protocol and the conventional (6 s) curing protocol. Based on the results obtained, the null hypothesis was partially rejected. Statistically significant differences in SBS were observed between the 3 s and 6 s curing groups when tested at 24 h (Groups 1 vs. 2, *p* = 0.002). However, when tested at 14 days, the difference between the 3 s and 6 s groups (Groups 3 vs. 4, *p* = 0.405) was not statistically significant, suggesting that post-cure polymerization over time may compensate for the shorter initial curing duration. Regarding ARI scores, the Kruskal–Wallis test revealed significant differences between the groups (*p* = 0.001), with longer curing times and extended storage periods associated with lower ARI scores, indicating that less adhesive remained on the enamel surface.

Bond strength testing is one of the most widely used and scientifically rigorous analyses in the evaluation of dental materials. This strength is measured in laboratory settings and serves as an indicator of material performance, which, in turn, helps predict clinical outcomes. The bond strength is quantified as the ratio of the force at which the bond fails (determined from the drop in load on the mechanical testing machine) to the surface area of the adhesive or bracket base. As we mentioned before, bonded brackets face various forces, including shear, tensile, and torsion forces, or combinations of these, making it challenging to accurately measure or quantify these forces [11,12].

Additionally, elements affecting the light-curing process of a resin-based composite should also be considered, such as the material’s composition (including opacity), the selection of photoinitiators, which can range from 0.1 to 1.0 percent by weight, and the concentration of these initiators [18]. Furthermore, the peak wavelength and bandwidth of the curing light, the light intensity, and the exposure time (duration of irradiation) also significantly impact the curing depth. Variables that may alter the amount of light energy delivered to the surface of a resin composite, possibly leading to insufficient polymerization, include the design and size of the light guide, the distance from the light-guide tip to the resin composite, the power density, the exposure time, the layer thickness, and the material composition [8,15].

However, in terms of the relationship between shear bond strength and curing time, several studies in the literature have documented a direct correlation between the increase in shear bond strength and curing time. Gronberg et al. (2006) demonstrated that increasing the curing time with an LED lamp leads to a statistically significant improvement in the shear strength of orthodontic brackets bonded to human enamel [19]. This study specifically evaluated the impact of both distance and exposure time when using a second-generation light-emitting diode unit, finding that an adequate curing time was essential for achieving optimal bond strength values. These findings align with the fundamental principles established by Rueggeberg et al. (1994), who demonstrated that both light intensity and exposure duration are directly correlated with the degree of cure of resin composites, which is closely related to a higher bond strength, since the material is better polymerized [6]. Their seminal work showed that an inadequate amount of light energy, whether from insufficient intensity or a short exposure time, leads to incomplete polymerization and compromised mechanical properties. Supporting the same idea, Lindberg et al. (2005) state that the more thoroughly cured the composite is at the enamel–adhesive-bracket interface, the higher the bond strength becomes, establishing a clear link between polymerization plenitude and clinical bond strength values [20,21]. Furthermore, Cacciafesta et al. (2005) demonstrated that the distance between the light-guide tip and the composite surface significantly impacts shear bond strength, reinforcing the principle that optimal light delivery and proper bonding techniques are essential for achieving adequate mechanical properties at the bracket–enamel interface [22]. Beyond the initial bonding performance, the long-term stability of resin-based materials is also influenced by their susceptibility to chemical and environmental challenges. Previous investigations have demonstrated that exposure to acidic and staining agents can induce surface degradation, microhardness reduction, and alterations in the chemical composition of resin composites, phenomena that may compromise their mechanical integrity and durability over time [23]. These findings on the relationship between bonding protocol quality and long-term mechanical stability reinforce the critical need for optimized light-curing parameters in all orthodontic applications. These collective findings emphasize that achieving optimal bond strength requires careful attention to multiple curing parameters, including light intensity, exposure time, and distance from the light source, as well as ensuring complete polymerization throughout the adhesive layer.

Beyond optimizing curing time and light intensity, the clinical application of these parameters requires careful consideration of additional variables that influence polymerization outcomes. The distance between the light-curing tip and the composite surface plays a critical role in determining the effectiveness of irradiance delivered to the adhesive layer. Diab et al. (2024) [15] demonstrated that exposure distance significantly affects light irradiance and that it also varies according to the operating modes of dental curing lamps. However, across all types and modes, increased distance results in substantial reductions in energy delivery. This finding is supported by earlier work from Cacciafesta et al. (2005), who showed that light-tip distance directly impacts the shear bond strength of composite resins, emphasizing the importance of maintaining optimal proximity during the curing procedure [22]. Furthermore, the relationship between adequate light energy delivery and polymerization quality has been extensively studied by Perković et al. (2023), who established a clear correlation between the degree of conversion and shear bond strength in orthodontic bonding procedures, demonstrating that incomplete polymerization at the adhesive–enamel interface compromises both bond integrity and long-term clinical stability [21]. This correlation is further validated by Lim et al. (2024) [24], who documented that both light irradiance and curing duration significantly impact the degree of conversion. They concluded that insufficient energy load leads to reduced polymerization, which is associated with inferior mechanical properties. However, clinicians must also consider the thermal effects of prolonged or high-intensity light exposure, as Duratbegović et al. (2024) reported that certain curing protocols can generate substantial temperature rises within the composite material, potentially causing patient discomfort or even pulpal damage if it is not properly managed [7]. Therefore, the optimal curing protocol should equilibrate aspects such as achieving complete polymerization, maintaining appropriate bond strength, minimizing chair time, and avoiding excessive heat generation that could compromise pulpal health.

The current study aimed to evaluate the influence of light intensity and curing time on the shear bond strength (SBS) and adhesive remnant index (ARI) of orthodontic brackets bonded with a light-cured adhesive system. The results showed that both parameters significantly influenced SBS and ARI scores. Longer curing times and higher light intensities generally resulted in higher bond strengths and less adhesive remaining on the enamel after debonding [21].

Our findings are in agreement with those reported by Gronberg et al. (2006) [19], as their research showed that increasing both light intensity and exposure time led to significantly higher SBS values. Their in vitro study also used a second-generation LED unit and demonstrated that both the curing distance and time play crucial roles in the polymerization effectiveness of orthodontic adhesives [8,10]. Moreover, our study showed similar results to Rueggeberg et al. (1994), who demonstrated that light intensity and exposure duration are directly correlated with the depth of cure and the final mechanical properties of resin-based materials [6].

In the present study, the highest SBS values were observed in groups with a 3200 mW/cm^2^ light intensity and 6 s curing time, particularly when samples were tested after 14 days. This supports the hypothesis that extended curing facilitates more complete polymerization, and that post-curing in artificial saliva can enhance the strength of the adhesive over time. The ARI scores were also significantly influenced, with higher light intensities and longer exposure times resulting in cleaner enamel surfaces post-debonding. Clinically, this could reduce chair time and the risk of enamel damage during adhesive removal [25].

Moreover, the lack of statistically significant difference between some 3 s and 6 s curing groups (e.g., Groups 3 and 4) suggests a possible plateau effect in polymerization efficiency beyond a certain exposure duration when using a moderate light intensity. This nuance emphasizes the need to balance the aspects mentioned before, such as curing time and light power, to achieve effective and efficient bonding, especially in time-sensitive orthodontic procedures [8,25].

## 5. Limitations

The present study has some limitations that should be considered when interpreting the results. First, as an in vitro investigation, the experimental conditions do not entirely replicate the complex oral environment, lacking several factors such as salivary flow, pH fluctuations, dietary influences, and the dynamic forces associated with mastication. Second, the variation in curing direction between groups (occlusal for 3 s groups vs. mesial/distal for 6 s groups) introduces a potential confounding variable. The uniformity and depth of adhesive polymerization may be influenced by the angle of light delivery. Although this protocol followed the manufacturer’s recommendations, future studies should aim to standardize the curing direction across all groups to identify the effect of curing time more precisely. Third, the sample was limited to extracted premolars, and results may not be directly applicable to other tooth types or to in vivo conditions. Fourth, only a single adhesive system (Transbond XT) was evaluated in this study; results may differ with other adhesive formulations or bonding protocols. Fifth, the relatively short aging periods (24 h and 14 days) may not fully reflect the long-term behavior of the adhesive bond under clinical conditions. Longer observation periods and thermocycling protocols would provide a more comprehensive assessment of bond durability. Sixth, shear bond strength testing represents a simplified mechanical model that applies a unidirectional force, whereas clinical bracket debonding involves complex, multidirectional force patterns. Finally, the degree of conversion of the adhesive was not directly measured; instead, shear bond strength and the ARI were used as indirect indicators of polymerization quality.

## 6. Recommendations for Further Investigation

Future studies incorporating degree-of-conversion analysis would provide a more comprehensive assessment of curing effectiveness. Subsequent investigations should also incorporate thermocycling protocols to simulate the thermal stresses that adhesive materials experience in the oral cavity, thereby providing a more clinically representative assessment of bond durability. In vivo studies or clinical trials are recommended to validate the present in vitro findings under actual clinical conditions, accounting for patient-related variables such as salivary contamination, occlusal forces, and oral hygiene practices. Finally, investigating a broader range of curing times (e.g., 1, 3, 6, and 10 s) and multiple LED intensities would help establish more precise clinical guidelines for optimal curing protocols in orthodontic bonding procedures.

## 7. Conclusions

Considering the limitations of the present in vitro study, the following conclusions could be drawn:

1. Light intensity and curing time significantly impact the shear bond strength (SBS) of orthodontic brackets bonded to enamel.
2. After 24 h, the 3 s curing protocol (3200 mW/cm^2^) produced significantly lower SBS values compared to the 6 s protocol, indicating that a longer curing time provides superior immediate bond strength.
3. After 14 days, the difference in SBS between the 3 s protocol and the 6 s protocol did not generate a statistically significant value (*p* = 0.405), suggesting that post-cure polymerization over time may partially compensate for the shorter initial curing duration. This, however, does not mean that 3 s and 6 s are equivalent in clinical practice, as early bond strength is critical for withstanding functional forces during treatment.
4. Lower ARI scores, indicating that less adhesive remained on the enamel surface, were found in groups cured with a higher intensity and longer duration, which may reduce the risk of enamel damage, plaque accumulation, and chair time during clean-up.
5. Optimizing curing protocols in clinical practice by creating a balance between all important aspects, such as curing time, light intensity, and clinical efficiency, could enhance bonding effectiveness and minimize clinical complications connected to bracket failure or excess adhesive remnants.

These findings support the use of high-intensity LED curing lights and adequate exposure times as a practical strategy for improving the outcomes of orthodontic bonding procedures.

## Figures and Tables

**Table 1:** Pairwise comparison of the shear bond strength between experimental groups (Tukey post hoc test *p*-values) (*n* = 20 per group).

	Group 1	Group 2	Group 3	Group 4
Group one		0.002	0.001	0.001
Group two	0.001		0.003	0.001
Group three	0.001	0.003		0.405
Group four	0.001	0.001	0.405	

**Table 2:** Adhesive remnant index (ARI) score distribution for each experimental group (*n* = 20 per group).

	No Adhesive = Score 0	<50% Adhesive = Score 1	>50% Adhesive = Score 2	100% Adhesive = Score 3	Mean Rank
Group one	3 15%	6 30%	6 30%	5 25%	50.00
Group two	4 20%	7 35%	7 35%	2 10%	44.15
Group three	6 30%	8 40%	3 15%	3 15%	38.73
Group four	9 45%	8 40%	3 15%	0 0%	29.13

## Data Availability

The data presented in this study are available from the corresponding author upon reasonable request.
